# Optimization of Properties for Alumina-Spinel Refractory Castables by CMA (CaO-MgO-Al_2_O_3_) Aggregates

**DOI:** 10.3390/ma14113050

**Published:** 2021-06-03

**Authors:** Hai Tang, Chunxue Li, Jianying Gao, Bruno Touzo, Chunfeng Liu, Wenjie Yuan

**Affiliations:** 1 The State Key Laboratory of Refractories and Metallurgy, Wuhan University of Science and Technology, Wuhan 430081, China; tanghaiwust@163.com; 2 Imerys Technical Center China, Tianjin 300450, China; chunxue.li@imerys.com (C.L.); simon.gao@imerys.com (J.G.); bruno.touzo@imerys.com (B.T.); chunfeng.liu@imerys.com (C.L.); 3 National-Provincial Joint Engineering Research Center of High Temperature Materials and Lining Technology, Wuhan University of Science and Technology, Wuhan 430081, China

**Keywords:** CMA aggregates, strength, slag resistance, thermal shock resistance, thermal fatigue resistance

## Abstract

Aiming at optimizing properties of alumina-spinel refractory castables, coarse corundum particles were replaced partially with the particles of a novel porous multi-component CMA (CaO-MgO-Al_2_O_3_) aggregate in the same size. Properties including the bulk density, apparent porosity, strength, slag corrosion resistance, thermal shock resistance and thermal fatigue resistance of alumina-spinel refractory castables containing CMA aggregates were evaluated contrastively. The results demonstrated that the incorporation of CMA aggregates can significantly improve thermal shock resistance and thermal fatigue resistance of castables, although companying with slight decrease in the bulk density and strength. Moreover, slag penetration resistance of castables can also be enhanced by CMA aggregates with appropriate particle size. The influence of CMA aggregates on properties of alumina-spinel refractory castables depended strongly on their particle size.

## 1. Introduction

Alumina-magnesia and alumina-spinel castables have been widely used in steel ladle linings below the slag zone, the well block of purging plugs, and injection lances due to their excellent slag resistance and mechanical properties [1,2]. In view of the increasing ratio of scrap to steel, higher working temperature, and prolonged refining time, the enhanced properties of materials, especially for the slag resistance and thermal shock resistance are required to adapt tougher service condition. In general, the slag penetration is reduced by using dense aggregates and designing the matrix with lower porosity through filling with fine powders. Nevertheless, the dense aggregates and matrix are hardly to release the thermal stress under the great temperature gradient. Regarding the practical applications, it is necessary to optimize the slag resistance and thermal shock resistance simultaneously. Recently, the exploitation of novel porous aggregates successfully broke the traditional concept, and it became a hot topic in refractories [3,4,5,6,7].

Extensive studies on porous aggregates have been carried out in recent years. The flexural strength of corundum-spinel castables changed only slightly, with the apparent porosity of aggregates increasing from 4.2% to 42% [8]. Regarding lightweight cordierite -mullite refractories, high strength and thermal shock resistance were achieved because of a superior interface bonding between the matrix and porous cordierite aggregates [9]. Yan et al. reported the penetration of slag towards matrix could be effectively inhibited with the introduction of porous aggregates [10]. Besides, the incorporation of porous aggregates can greatly reduce the thermal conductivity of materials, which can raise the energy efficiency of high temperature containers [10,11].

Although, the porous aggregates have enormous advantages compared with conventional one as above mentioned. At present, the sort of lightweight aggregates used in the steel ladles are often referred to as lightweight corundum and periclase-spinel aggregates [12,13]. However, developing other multicomponent lightweight aggregates provided an alternative for the design and applications of refractories, i.e., CMA (CaO-MgO-Al_2_O_3_) aggregates mainly comprised with spinel, CA (CaAl_2_O_4_) and CA_2_ (CaAl_4_O_7_). The slag resistance of magnesia-carbon (MgO-C) bricks for steel ladle linings was significantly improved with CMA aggregates addition as a consequence of an in situ formation of a protective layer on the surface of bricks [14,15]. The corrosion mechanism of alumina-spinel castables with CMA aggregates addition has been demonstrated by Wöhrmeyer et al. The results indicated that a thin densified zone formed and blocked the slag penetration into the porous matrix and the porous aggregates [16]. However, the comprehensive performance of castables was critical for the applications. Considering aggregates may have great influence on various aspects of performances of castables, CMA aggregates with different particle sizes were selected in this study. The influence on properties, especially the slag resistance and thermal shock resistance of alumina-spinel castables, was investigated. The aim of this work was therefore to optimize properties of alumina-spinel refractory castables through incorporating CMA aggregates.

## 2. Materials and Methods

All specimens were formulated by the compositions listed in Table 1. White fused alumina (Sanmenxia) and tabular alumina (Almatis) served as the source of Al_2_O_3_. A portion of spinel (AR78, Almatis) and reactive alumina (BMP, Higiant) were used in this study. CMA72 cement (Imerys) acted as the binder of castables. CMA aggregates (MagArmour^®^, Imerys, Paris, France) with various sizes (1–3, 3–5 and 5–8 mm) were selected to replace the alumina aggregates with corresponding sizes, respectively, and the addition of CMA aggregates was 8 wt% in all samples. With the function of water reducing agents REFPAC288 and REFPAC388B (Imerys, Paris, France), the range of water demand for casting was 4.1–4.6%.

**Figure 1:**
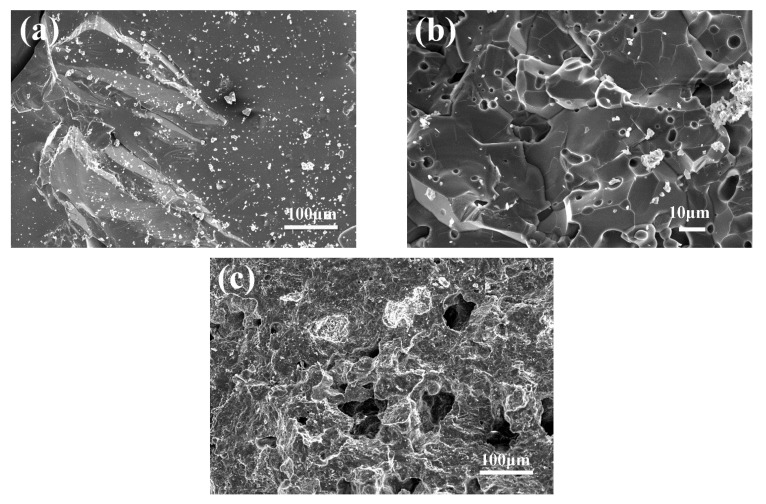
Microstructure of three aggregates: (**a**) white fuse alumina, (**b**) tabular alumina and (**c**) CMA aggregates.

The major phase of CMA aggregates included spinel (MA), CA and less amount of CA_2_ as listed in Table 2. The bulk density (2.3 g/cm^3^) of CMA aggregates was obviously lower than white fused alumina (3.9 g/cm^3^) and tabular alumina (3.6 g/cm^3^). Microstructure of three types of aggregates was presented in Figure 1. Extremely dense grains of fused alumina were observed (Figure 1a). Compared with fused alumina, there were more pores in tabular alumina with smaller grain size as shown in Figure 1b. Larger pores were distributed in porous CMA aggregates with an apparent porosity of around 28% (Figure 1c), which resulted in lower bulk density than the other two types of aggregates.

After casting, all castables were cured under the conditions of 20 °C and 70–75% relative humidity for 48 h, then dried at 110 °C for 24 h. All samples were calcined at 1100 °C and 1550 °C for 3 h, respectively. The apparent porosity and bulk density of samples were measured by the Archimedes technique using the GB/T 2997-2000 standard [17]. The cold modulus of rapture (CMOR) was measured with three-point bending tests following GB/T 3001-2007 [18]. The phase composition of castables was characterized by X-ray diffraction (XRD, X’pert Pro MPD, Philips, Almelo, The Netherlands). Microstructure of specimens were characterized by scanning electron microscopy (SEM, JEOL JSM-6610, JEOL Ltd., Tokyo, Japan). The slag resistance experiment of samples was carried out by adopting the static crucible method following GB/T 8931-2007 [19]. The chemical composition of slag was listed in Table 3. After heating at 1550 °C for 3 h, samples were cut open along the center line to observe the status of the corrosion. The corrosion and penetration index were calculated by the equations I_c_ = A_cl_/A_c_ × 100% and I_p_ = A_pl_/A_c_ × 100%, where A_cl_ is the area of sample corroded by slag, A_pl_ is the area of sample penetrated by slag, and A_c_ is the area of the crucible. The thermal shock resistance was tested by air quenching method (5 cycles) according to YB/T 2206.1-1998 [20]. The samples firstly were put in an electric furnace soaking at 1100 °C for 30 min, then were cooled down to room temperature by the compressed air. After each thermal cycle, the elastic modulus of samples was measured by Elastic Modulus & Damping System (RFDA, HTVP1600, IMCE, Genk, Belgium). Additionally, the residual strength and elastic modulus of specimens after 5 cycles were measured to evaluate the thermal shock resistance (TSR) and thermal fatigue resistance (TFR) of samples.

## 3. Results

### 3.1. Phase Composition

**Figure 2:**
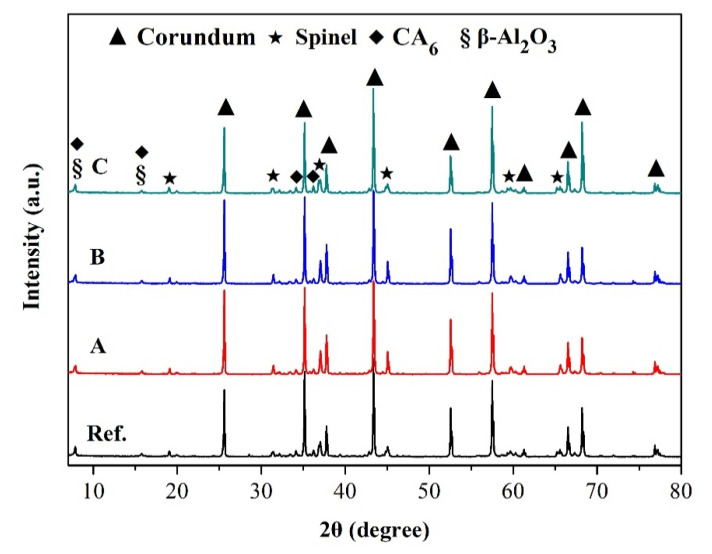
XRD patterns of castables after firing at 1550 °C for 3 h.

XRD patterns of specimens calcined at 1550 °C for 3 h are presented in Figure 2. Castables were mainly comprised with corundum and spinel. Additionally, a little amount of CA_6_ as the product between cement and alumina powders was detected. The formation of CA_6_ can result in a certain volume expansion (3.01%), and its plate structure was beneficial to the strength of materials [21,22]. The industry alumina manufactured by Bayer process usually contained less impurity Na_2_O [23], thus the peaks of β-Al_2_O_3_ (Na_2_O·11Al_2_O_3_) were also observed in Figure 2. The similar chemical composition of castables determined the difference of phase compositions among these samples was limited. Therefore, the influence of phase composition on the properties of castables was ignored in the following discussion.

### 3.2. Microstructure

**Figure 3:**
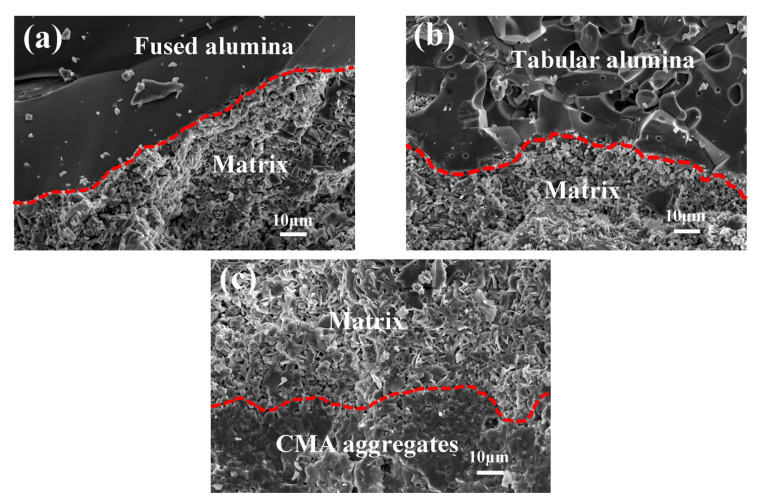
Microstructure of the bonding interfaces between the matrix and aggregates in castables calcined at 1550 °C for 3 h: (**a**) Fused alumina, (**b**) tabular alumina and (**c**) CMA aggregates.

Figure 3 shows the bonding interface between aggregates and matrix of samples calcined at 1550 °C for 3 h. The edges of alumina particles acted as the interface between aggregates and matrix as seen in Figure 3a,b. However, the interface between matrix and CMA aggregates was not so easy to identify as shown in Figure 3c. It was generally assumed that the porous aggregates had larger specific surface areas than conventional dense aggregates, so higher sintering activity contributed to this feature. Besides, the pores at the surface of porous aggregates can be filled by fine powders during the preparation process of castables and finally formed an interlocking structure after sintering [10]. As listed in Table 2, CMA aggregates contained CA and CA_2_, which can further react with alumina powders in the matrix to reinforce the bonding of interfaces. The above mentioned factors resulted in the better combination for interface between CMA aggregates and the matrix, as seen in Figure 3c.

### 3.3. Physical and Mechanical Properties

In Table 4, the physical and mechanical properties of castables such as the bulk density, apparent porosity and strength are listed. The apparent porosity of the reference sample was relatively lower, and its bulk density was slightly higher than others. Meanwhile, the apparent porosity of samples presented an increasing tendency with temperature rising from 1100 °C to 1550 °C considering the expansive effect of CA_6_ formation [22]. Moreover, small differences for the strength of samples were presented after castables calcined at 1100 °C, cold compressive strength (CCS) and cold modulus of rupture (CMOR) of castables containing CMA aggregates were reduced by 9% and 22.5%, respectively, compared to the strength of reference sample calcined at 1550 °C. Normally, the fracture of materials under the load was the result of cracks extension. Three mechanisms of the crack propagation were usually mentioned, which were cracks extended through aggregates, matrix or along the interface between aggregates and matrix [6]. It was assumed that the bonding strength of interfaces and the densification of matrix in samples were limited after being calcined at 1100 °C. In this case, cracks preferred to propagate along the interface and matrix rather than through aggregates. Therefore, the porous microstructure and the particle size of CMA aggregates had little effects on the strength of castables calcined at this temperature. On the opposite, the aggregates would suffer more load considering the sintering degree of the matrix and the bonding strength of interfaces were intensified at a higher sintering temperature of 1550 °C. The strength of conventional dense aggregates (fused alumina and tabular alumina) was higher than CMA porous aggregates due to compact microstructure and well-developed crystals of the former as presented in Figure 1. However, strong refractories were not always suitable. It was stated that the lower brittleness could be mainly achieved by a decrease of strength [24]. Besides, it was pointed out that pores in aggregates could help to arrest the crack propagation through the grains and grain boundary in alumina-based refractory [25], therefore aggregates played an important role to resist the cracks extension as well as the thermal shock failures, which will be discussed in a later section.

### 3.4. Slag Corrosion Resistance

**Figure 4:**
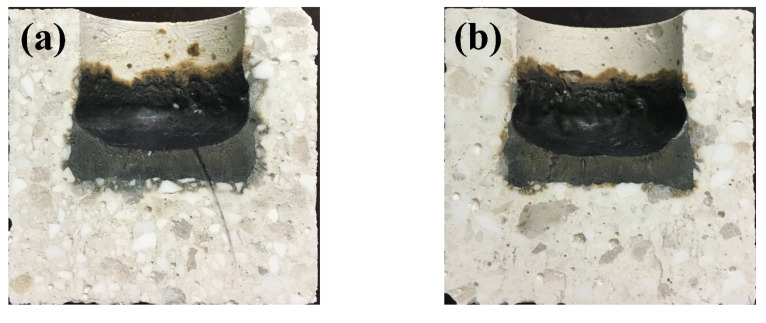

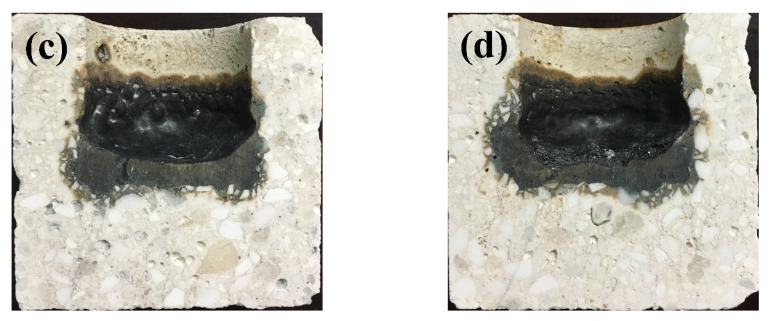
Corroded specimens after the corrosion test: (**a**)-Ref., (**b**)-A, (**c**)-B and (**d**)-C.

**Figure 5:**
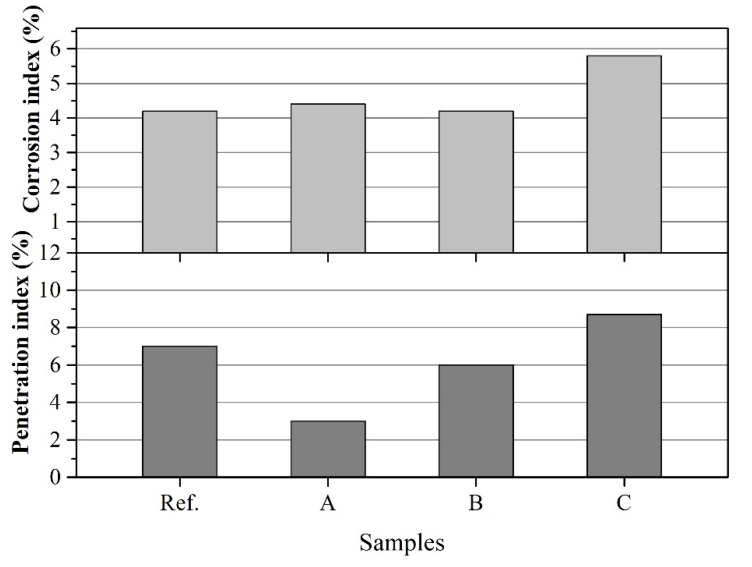
Corrosion and penetration index of corroded specimens after the corrosion test.

The photographs of corroded specimens are given in Figure 4. The calculated corrosion and penetration index are presented in Figure 5. It was easily observed that the border between slag and castables of sample A was much flatter as shown in Figure 4. Although there was no remarkable difference of corrosion index among samples reference, A and B, the penetration index of sample A was lower than that of other samples as shown in Figure 5. Moreover, the slag resistance of castables gradually declined with the increasing of the size of CMA aggregates, especially for sample C. Thus, the incorporation of CMA aggregates with appropriate size can enhance the slag resistance of castables. Simultaneously, the effects were directly correlated with their particle size.

The slag corrosion resistance of castables depended on the dissolution rate of materials into the slag and the spalling of the deteriorated layer [26]. Although the dissolution characteristic of each phase in the slag was different, the spalling of the deteriorated layer caused by the penetration of slag was more likely to trigger the damage of refractories [27]. Hence, the penetration index was one of the important factors to evaluate the slag resistance of castables. It was indicated that the complex phase composition of CMA aggregates was beneficial to form calcium aluminum silicate glass phase, which can prevent slag from deep penetration in refractories [14,15,16]. This mechanism was mainly concerned the chemical composition of CMA aggregates. However, the porous microstructure was another key characteristic for CMA aggregates unlike conventional dense aggregates. Theoretically, the slag penetrated in the matrix was under the action of capillary force. When the molten slag came into CMA aggregates, the porous structure of aggregates can absorb the slag by the capillary force, resulting a smaller depth of penetration layer and less liquid formed considering lower concentration of the slag in the matrix [10]. The smaller particles had the advantages such as the uniform distribution and high specific surface area than the bigger one. For the larger particles, it was harder to absorb the slag into its porous structure from the matrix in view of less likely to contact the slag. This probably explained the higher penetration index of samples containing CMA aggregates with larger size as shown in Figure 5.

Spinel containing castables present an outstanding slag resistance due to the high melting point and chemical stability of spinel in the slag, i.e., spinel does not react with the infiltrating slag [28]. According to the phase composition of CMA aggregates as listed in Table 2, CMA aggregates were mainly comprised with spinel (73.2 wt%). This meant CMA aggregates was a reinforced phase to resistance the corrosion of slag. If too big CMA aggregates (5–8 mm) added in castables, it would result a higher corrosion rate considering spinel almost located at the inner of CMA aggregates and hardly prevented the slag corroded the matrix. This can be accounted for the higher corrosion index of sample C than sample A and B as presented at Figure 5.

### 3.5. Thermal Shock Resistance

**Figure 6:**
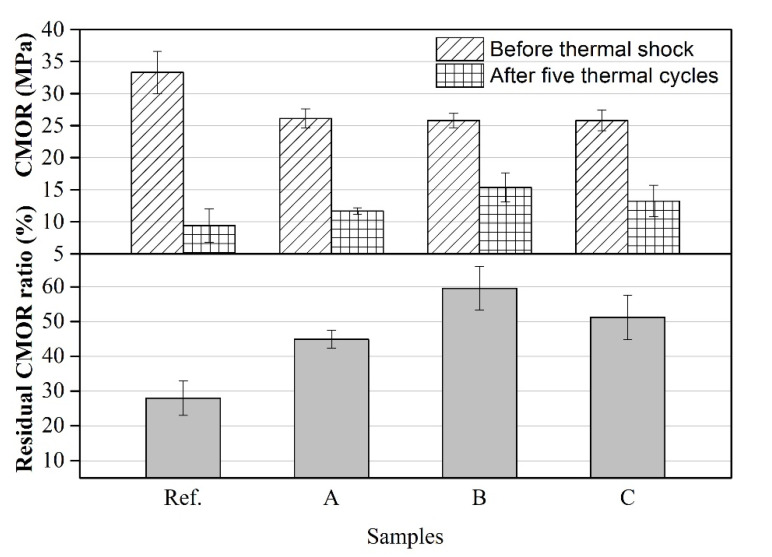
Variation of cold modulus of rupture (CMOR) and residual CMOR ratio of samples after 5 thermal cycles.

Thermal shock resistance (TSR) of refractory castables is closely associated with raw materials and microstructure [29]. It has been verified that the utility of high strength for thermal shock resistance was not a good practice [25]. Hence, the raw material selection and refractory design is a critical concern for practical applications. In order to study the influence of CMA aggregates on the thermal shock resistance of castables, CMOR and residual CMOR ratio of samples after air quenching test (5 times) were measured and calculated, respectively. As presented in Figure 6. Both residual CMOR and its ratio for reference samples were dramatically lower than the others. Moreover, the residual CMOR ratio of sample B reached 60% after five thermal shock cycles. By comparison, this value for the reference sample was only 28%. It was demonstrated that the stress concentration was more likely to occur at the interface between matrix and aggregates in consideration of their different thermal expansion coefficients when suffering thermal shock [30]. The better interface combination above mentioned can provide higher strength to resistance of the thermal stress. In addition, the thermal expansion and shrinkage caused by the temperature changing can be remitted with the function of micropores in porous aggregates, and finally the stress concentration was effectively reduced [31]. These factors led to better thermal shock resistance of castables containing CMA aggregates.

### 3.6. Thermal Fatigue Resistance

**Figure 7:**
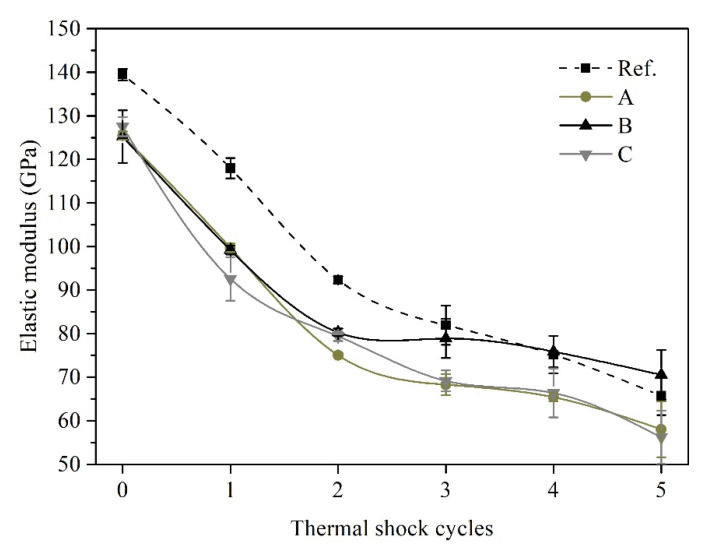
The variation of elastic modulus of castables with thermal shock cycles.

The evolution of phase and microstructure as well as the microcrack formation had a major impact on the elastic modulus (E) of refractories [32]. As is known to all, the drastic temperature change can result in the damage of microstructure, i.e., the initiation and propagation of cracks, and this was reflected by the decreasing of the value of E. The variation of elastic modulus of castables with thermal shock cycles was corresponding to the changes of properties as shown in Figure 7. The decline of elastic modulus for castables after the first two cycles was remarkable, then the extent of reduction relatively decreased during three to five thermal shock cycles. Similar phenomena were observed for alumina-based and mullite-based refractory castables [33]. The residual elastic modulus of sample B was greater than that of the reference sample after five thermal shocks, which was consistent with strength data in Figure 6.

E and E_0_ refer to the elastic modulus after and before the thermal shock, respectively. The ratio E/E_0_, namely TFR (thermal fatigue resistance), can also be regarded as an indicator of the thermal behavior of refractory materials. The relation between the ratio E/E_0_ and thermal shock cycles (N) followed the equation as below: [34]

E/E_0_ = *a* + (1 − *a*) e ^(−*b* N)^ (1)

when N is large enough, the value of E/E_0_ stays a constant, namely the parameter *a* according this equation. The value of parameter *a* and *b* is useful for quantitatively comparing TRF. Basically, a higher value of parameter *a* means a better TFR at saturation stage, and a lower value of *b* means the better resistance to thermal degradation at the initiation stage [34].

**Figure 8:**
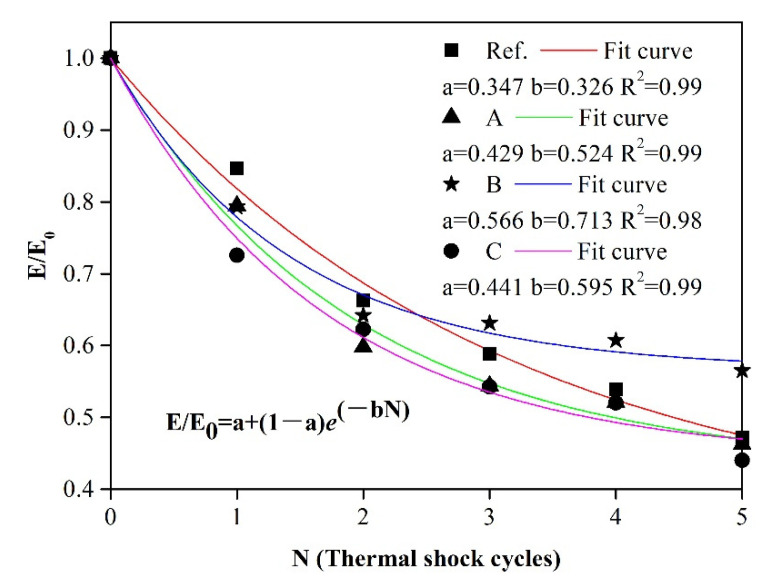
Thermal fatigue resistance (TFR) behavior of castables.

Figure 8 shows the relation E/E_0_ as a function of the number of quenching tests. The experimental data for all specimens was fitted with the above equation and also plotted in Figure 8. The compelling fitting results were obtained with the coefficient of determination R^2^ ≥ 0.98. The parameter *b* of reference sample was least, which was in agreement with higher E/E_0_ ratio of reference sample after the first two cycle than others. However, the samples with CMA aggregates addition presented better TFR than reference sample especially for specimens B in view of the value of parameter *a*, which was consistent with the result of TSR.

In general, the numerous cracks formed at initial stage (the first several times thermal shock cycles) following with the dramatical decreasing of E for materials. Then, more energy deriving from thermal shock was needed for further extension of cracks and the formation of new cracks. When the thermal stress was not enough for the development of cracks, the value of E remained almost a constant with the next thermal shock treatment. This stage was called saturation stage [35]. The fitted curve (E/E_0_) of reference sample presented a continuous decreasing tendency. In comparison, samples containing CMA aggregates reached the saturation stage after two or three cycles.

The incorporation of CMA aggregates can improve the thermal shock resistance and thermal fatigue resistance of alumina-spinel refractory castables as described above. It could be found that the samples containing 3–5 mm CMA aggregates presented best thermal shock resistance. Although the initiation resistance of castables containing CMA aggregates was slightly worse, they presented a superior thermal fatigue resistance after two cycles. This mechanism needs to be investigated further.

## 4. Conclusions

Compared to the conventional dense corundum aggregates, porous CMA (CaO-MgO-Al_2_O_3_) aggregates comprised with spinel, CA and CA_2_ had a strong impact on properties of alumina-spinel refractory castables. The following aspects were evaluated. The partial substitution of conventional aggregates with CMA aggregates slightly reduced the bulk density and strength of alumina-spinel refractory castables to a certain extent. The bonding of interfaces between aggregates and the matrix was enhanced due to higher activity of CMA. The performance of castables depended on the particle size of CMA aggregates. More uniform distribution and porous structure of smaller CMA aggregates with 1–3 mm presented the best slag penetration resistance. The residual CMOR ratio of castables containing 3–5 mm CMA aggregates after five cycles of thermal shock treatment increased twofold compared to the reference. Regarding higher retained elastic modulus ratio, castables containing 3–5 mm CMA aggregates had a superior thermal fatigue resistance. Castables containing CMA aggregates with a low initial elastic modulus reached the saturation stage after two or three cycles. The use of CMA aggregates offered an alternative to optimize properties of alumina-spinel refractory castables.

## Figures and Tables

**Table 1:** Compositions of alumina-spinel refractory castables.

Raw Materials	Content (wt%)			
	Ref.	A	B	C
White fused alumina(5–8 mm)	15	15	15	7
Tabular alumina (3–6 mm)	17	17	9	17
Tabular alumina (1–3 mm)	20	12	20	20
Tabular alumina (≤1 mm)	25	33	33	33
Spinel (≤1 mm)	8	0	0	0
Reactive alumina (BMP)	5	5	5	5
Cement (CMA72)	9	9	9	9
CMA aggregates (1–3 mm)	0	8	0	0
CMA aggregates (3–5 mm)	0	0	8	0
CMA aggregates (5–8 mm)	0	0	0	8
Additives (REFPAC288 and REFPAC388B)	1	1	1	1

**Table 2:** Mineralogy of CMA aggregates.

Phase	MA	CA	CA_2_	Others
Content (wt%)	73.2	16.9	7.8	2.2

**Table 3:** Chemical compositions of slag used for testing.

Compositions	CaO	Fe_2_O_3_	SiO_2_	MgO	Al_2_O_3_	Others
Content (wt%)	43.8	29.9	11.8	5.4	3.6	5.5

**Table 4:** Apparent porosity, bulk density, CCS and CMOR of samples calcined at different temperatures.

Temperature	No.	Apparent Porosity (%)	Bulk Density (g/cm^3^)	Cold Compressive Strength (MPa)	Cold Modulus of Rupture (MPa)
1100 °C	Ref.	14.6 ± 0.3	3.16 ± 0.01	91.2 ± 7.1	10.1 ± 1.1
	A	15.9 ± 0.1	3.10 ± 0.01	87.2 ± 6.6	10.0 ± 1.7
	B	15.8 ± 0.6	3.10 ± 0.02	87.6 ± 2.1	10.2 ± 1.3
	C	15.7 ± 0.3	3.09 ± 0.02	86.0 ± 1.8	10.4 ± 0.1
1550 °C	Ref.	15.4 ± 0.1	3.18 ± 0.01	220.2 ± 0.2	33.3 ± 3.3
	A	16.8 ± 0.3	3.11 ± 0.01	206.1 ± 1.1	26.1 ± 1.5
	B	16.4 ± 0.1	3.11 ± 0.01	200.7 ± 1.9	25.8 ± 1.1
	C	16.7 ± 0.2	3.09 ± 0.01	200.6 ± 4.4	25.8 ± 1.6

## Data Availability

The data are not publicly available.
